# Optimization of native biocontrol agents, with parasitoids of the invasive pest *Drosophila suzukii* as an example

**DOI:** 10.1111/eva.12648

**Published:** 2018-06-14

**Authors:** Astrid Kruitwagen, Leo W. Beukeboom, Bregje Wertheim

**Affiliations:** ^1^ Groningen Institute for Evolutionary Life Sciences University of Groningen Groningen The Netherlands

**Keywords:** artificial selection, biological control agent, coevolution, exotic species, host–parasite interactions, pest management, phenomics, spotted wing *Drosophila*

## Abstract

The development of biological control methods for exotic invasive pest species has become more challenging during the last decade. Compared to indigenous natural enemies, species from the pest area of origin are often more efficient due to their long coevolutionary history with the pest. The import of these well‐adapted exotic species, however, has become restricted under the Nagoya Protocol on Access and Benefit Sharing, reducing the number of available biocontrol candidates. Finding new agents and ways to improve important traits for control agents (“biocontrol traits”) is therefore of crucial importance. Here, we demonstrate the potential of a surprisingly under‐rated method for improvement of biocontrol: the exploitation of intraspecific variation in biocontrol traits, for example, by selective breeding. We propose a four‐step approach to investigate the potential of this method: investigation of the amount of (a) inter‐ and (b) intraspecific variation for biocontrol traits, (c) determination of the environmental and genetic factors shaping this variation, and (d) exploitation of this variation in breeding programs. We illustrate this approach with a case study on parasitoids of *Drosophila suzukii*, a highly invasive pest species in Europe and North America. We review all known parasitoids of *D. suzukii* and find large variation among and within species in their ability to kill this fly. We then consider which genetic and environmental factors shape the interaction between *D. suzukii* and its parasitoids to explain this variation. Insight into the causes of variation informs us on how and to what extent candidate agents can be improved. Moreover, it aids in predicting the effectiveness of the agent upon release and provides insight into the selective forces that are limiting the adaptation of indigenous species to the new pest. We use this knowledge to give future research directions for the development of selective breeding methods for biocontrol agents.

## 1 INTRODUCTION

Invasive pest species are a worldwide problem and can cause high economic losses when they feed on economically important crops (Aukema et al., 2010; Oliveira, Auad, Mendes, & Frizzas, 2013; Pimentel, Zuniga, & Morrison, 2005; Pimentel et al., 2001). An example of such invasive pest is the Spotted Wing *Drosophila*,* Drosophila suzukii* (Species authority can be found at eol.org). This Asiatic fruit fly has invaded Europe and North America since 2008 (Calabria, Máca, Bächli, Serra, & Pascual, 2012; Cini, Ioriatti, & Anfora, 2012; Hauser, 2011) and causes large economic damage to a wide range of soft and stone fruits (Bolda, Goodhue, & Zalom, 2010; De Ros, Anfora, Grassi, & Ioriatti, 2013; Goodhue, Bolda, Farnsworth, Williams, & Zalom, 2011; Walsh et al., 2011). To suppress exotic pest populations such as *D. suzukii*, there is a growing interest to develop environmental friendly managing methods to reduce the application of harmful pesticides. A traditional nonchemical method is biological control: the release of a pest's natural enemy to suppress its population. This method has been proposed as the best pest management strategy for maximizing environmental safety and economic profitability (Cock et al., 2010; van Lenteren, 2012b) and is often used in combination with other strategies (e.g., mass trapping, sanitation, crop rotation) as part of an integrated pest management (IPM) approach (Cock et al., 2010).

To develop a biocontrol managing strategy, a control agent should be chosen that is highly efficient at suppressing the pest population growth. Exotic pest species, however, have (initially) no or only a limited number of natural enemies in the invasive area, as these indigenous natural enemies present in the invasive range are not (yet) adapted to the pest. This also applies to *D. suzukii* as it has only few species of natural enemies in the invasive area compared to its area of origin (Asplen et al., 2015; Chabert, Allemand, Poyet, Eslin, & Gibert, 2012; Miller et al., 2015; Nomano, Mitsui, & Kimura, 2015). Therefore, it is common practice to import and release natural enemies from the native range of the pest, as they are more efficient due to their long coevolutionary history with the pest. Biodiversity risks (De Clercq, Mason, & Babendreier, 2011; Hajek et al., 2016) and new international regulations; in particular, the Nagoya Protocol on Access and Benefit Sharing (Cock et al., 2010; Hajek et al., 2016; van Lenteren, 2012b), however, currently limit the use of exotic natural enemies and challenge the development of biocontrol for alien pest species. Although these regulations are vital for the protection of native species, they also restrict the number of species available for biological control (van Lenteren, 2012b; van Lenteren, Bolckmans, Köhl, Ravensberg, & Urbaneja, 2018). These factors often lead to the use of less fit indigenous rather than well‐adapted exotic natural enemies for new biological pest management strategies. Hence, there is a strong need to develop methods to improve indigenous natural enemies to increase their efficiency and safety for managing exotic pest species.

According to tradition, agents are chosen based on interspecific variation (variation between species), using those species that seem best at controlling the pest in the target area (van Lenteren, 2012a; Lommen, de Jong, & Pannebakker, 2017). However, this has resulted in a highly variable success rate (Collier & Van Steenwyk, 2004) and may not meet the number of control agents needed in the future (Lommen et al., 2017). A promising approach is to exploit natural genetic intraspecific variation (variation within species) to improve control agents, by selecting and breeding only those individuals of a candidate species with the desired characteristics (Lommen et al., 2017). Intraspecific variation can be used in two ways: (a) choosing the most competent strain (“strain selection”) for biocontrol and (b) selecting only those individuals from population(s) with desired traits to form the parents of the next generation (“selective breeding” or “artificial selection”). Surprisingly, although this has been proposed in the literature repeatedly (Hopper, Roush, & Powell, 1993; Hoy, 1986) and has been widely applied in traditional agriculture (e.g., plant and animal breeding), only a limited number of researchers have taken this approach to biocontrol agents (Hoy, 1986; Lommen et al., 2017). Novel genetic techniques are also being developed, such as RNA interference, CRISPR/Cas genome editing and Release of Insects with Dominant Lethals (RIDL) (Leftwich, Bolton, & Chapman, 2016). Although these techniques show great potential, they currently cannot be widely applied due to GMO regulations and the perceived high ecological risks (Kolseth et al., 2015; Vàzquez‐Salat, Salter, Smets, & Houdebine, 2012; Webber, Raghu, & Edwards, 2015). Selective breeding, on the other hand, is an environmentally safe and socially accepted method.

**Figure 1:**
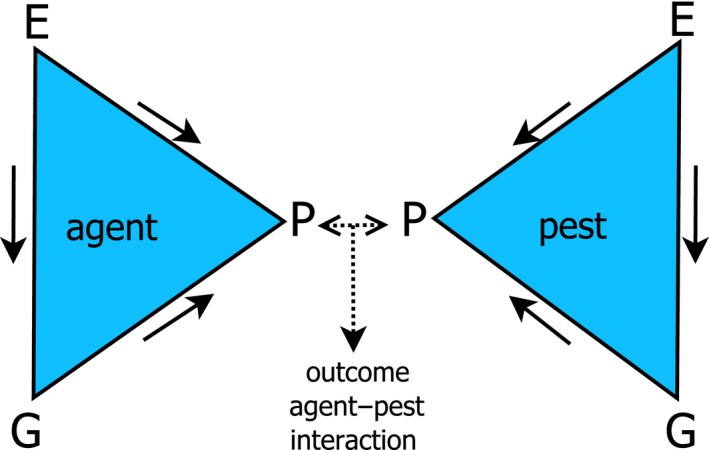
Sources of variation that determine the outcome of the agent–pest interaction: death of the pest, the agent, the pest and the agent, or the survival of both. The factors leading to this variation include heritable and nonheritable sources. P = phenotypic variation of the agent and pest; G = heritable variation consisting of genetic and epigenetic variation of the agent and pest; E = environmental source of variation affecting the agent and the pest. Some aspects of this environment are perceived by both (e.g., temperature and pesticides), while other aspects may concern only the pest or agent (e.g., abundance of alternative host species). Arrows indicate interaction between sources of variation: environmental and (epi‐)genetic sources affecting the phenotype directly or environmental conditions affecting the genotypic expression (phenotypic plasticity)

Optimization of traits important for biocontrol via selective breeding requires presence of heritable genetic variation. Variation and expression of traits can, however, also be due to environmental variation (phenotypic plasticity) and/or variation in how genotypes respond to environmental change (genotype [G] × environment [E] interaction) (Figure 1). This phenotypic plasticity may impede trait optimization across different environments, while the extent of phenotypic plasticity can be heritable. In an interesting manner, this information can also be exploited in selective breeding for a specific target area of release in case there is a strong G × E interaction. For example, agents can be selected for robustness (high performance in the range of relevant environmental conditions) or one can introgress alleles in the agent enabling adaptation to the target area of release (Hayes, Daetwyler, & Goddard, 2016). Moreover, the success of a control agent is also influenced by the phenotype of the pest (Figure 1) (e.g., larval feeding depth and thus accessibility to parasitization (Meijer, Smit, Schilthuizen, & Beukeboom, 2016)). The interaction of the control agent with the pest can have variable outcomes, death of the pest and/or the agent, which depends on the genetic and environmental factors they both encounter. In other words, the success of the agent depends on the genotype‐by‐genotype‐by‐environmental interaction (G_h_ × G_p_ × E) (Agrawal, 2001; Thomas & Blanford, 2003). It is therefore important to understand the genetic as well as the environmental factors that influence the phenotype of the agent for its success to suppress a specific pest population in the area of release. Optimization not only includes the use of heritable variation (selective breeding) but can also act on nonheritable variation. For example, altering specific environmental conditions by additional management strategies can weaken the pest population, which makes it more susceptible to the control agent, and this increases the killing efficiency of the agent. Moreover, the killing efficiency of the agent can also be influenced by experience of the agent with the pest; in particular, parasitoids show a learning ability that may increase their killing efficiency. Learning ability therefore can also be used to optimize biocontrol agents (Giunti et al., 2015). Hence, optimization can also rely on nonheritable sources of variation (e.g., learning of certain stimuli).

In this review, we address the question: How can evolutionary biology principles be used to improve native natural enemies for their use as biocontrol agent, by exploitation of their intraspecific trait variation? We mainly focus on selective breeding, but also indicate additional approaches, including exploitation of learning ability during breeding and manipulation of environmental conditions in the area of release to enhance the impact of the biocontrol agent. To fully appreciate the potential of selective breeding, we first propose a four‐step approach in which we underline the importance of an in‐depth understanding of those traits that determine the performance of a potential agent, both its ability to suppress the pest population in the target area and its amenability to mass rearing. This includes the investigation of the genetic variation and heritability of the trait of interest, and how this can be exploited, as described by Lommen et al. (2017). In addition, we show that besides genetic factors, knowledge of biotic and abiotic factors that affect the interaction between the biocontrol agent and the pest is crucial for optimization. We illustrate this approach with a case study on the new invasive pest *D. suzukii* and its important natural enemies, parasitoids. Development of environmental friendly management methods is urgently needed for this major pest in Europe and North America because, at the moment, the main control method is large‐scale pesticide use (Asplen et al., 2015; Bruck et al., 2011; Cini et al., 2012; Haye et al., 2016; Timmeren & Isaacs, 2013). Based on the four‐step approach and a review of knowledge about *D. suzukii*–parasitoid interactions, we show how the performance of indigenous parasitoids in the invasive area can be optimized for biocontrol. We will not review the different methods of selective breeding as this has been recently covered by Lommen et al. (2017). We also suggest future research directions for improvement of biocontrol agents.

## 2 IMPROVEMENT OF NATURAL ENEMIES BY EXPLOITING NATURAL VARIATION: A FOUR‐STEP APPROACH

To improve the performance of potential indigenous control agents against an invasive pest, first the most promising natural enemies have to be chosen for optimization. They should be selected based on traits enabling high biocontrol performance, that is, efficient (large‐scale) production and significant pest population reduction in the target area. These “biocontrol traits” include high killing efficiency, robustness under (a)biotic conditions in the area of release, environmental safety, and ability to be cost‐effectively (mass) reared in the laboratory (Table 1). It should be recognized that many of the biocontrol traits actually comprise multiple aspects of the behavior and physiology of the agent. For example, high killing efficiency of a parasitoid may rely on the adequate localization of host habitats, host‐finding, host recognition and acceptance, sufficient fecundity, and high parasitization success rate (Fleury, Gibert, Ris, & Allemand, 2009) (Table 1).

**Table 1:** List of biocontrol traits that determine the performance of a (potential) biocontrol agent

Biocontrol traits that determine performance	Example of species trait values that determine performance	Example of species trait values of parasitoids of *Drosophila suzukii* that determine performance
High killing efficiency in area of release	Host localization ability, finding large part of the pest population	Localize *D. suzukii* in ripening soft fruits on trees/plants and (in) fallen (fruits) on the ground, long ovipositor to reach larvae inside fruits
	High attack rate (preferably during entire lifetime)	Large number of mature eggs available (egg load), high oviposition rate
	High killing success rate of individual agents, such that a large part of the pest population is killed	Ability to suppress host immune response, kill *D. suzukii* larvae/pupae
	Prefer pest species over alternative prey/host	Preference for *D. suzukii* over other host (*Drosophila*) species
	Low dispersal tendency from patch/microhabitat of the pest (if pest is patchily distributed)	Stay in fruit patch until all *D. suzukii* larvae/pupae have been parasitized
	Low dispersal from agricultural habitat (for long‐term control: persist in the area also at low pest density)	Limited long‐distance dispersal (e.g., <50–100 m), and (for ongoing control) use of alternative host species at low *D. suzukii* density
	Density responsiveness	Locate larvae/pupae at low *D. suzukii* density, increase oviposition rate with increasing *D. suzukii* density
	Recognize suitable host/prey	Ability to recognize already parasitized hosts (avoidance super‐/multiparasitism), in particular when eggs are limited and for long‐term control when supernumerary eggs result in death of the agent
	Able to efficiently kill pest population in target area (requires insight into potential intraspecific differences between pest populations)	Able to overcome immune resistance of *D. suzukii* population in target area (requires insight into amount of intraspecific variation in immunity of *D. suzukii*)
	For ongoing control: able to build up and maintain a population over multiple generations	Complete entire life cycle on *D. suzukii* (survive parasitization of *D. suzukii* larvae or pupae*)*, finding of suitable mates, ability of adults to find food
Robustness under (a)biotic conditions in area of release	High fitness at climatic conditions in area of release (survival, high killing efficiency). Depends on, for example, target crop whether it is growing outside and vulnerable to precipitation and unpredictable weather conditions or more stable climatic conditions in greenhouse	Survival and high killing efficiency at relative low or high temperature (e.g., 15–20°C/>25°C) when released early or late in growing season and/or at high/low humidity
	High fitness (survival, high killing efficiency, activity) at timing of release (early/mid/late in growing season) and during aimed duration of control (1 or more generations during one or multiple seasons)	Low sensitivity to variable climatic conditions throughout the year (for long‐term control)
	Low sensitivity to agricultural practices in area of release	Tolerant to crop manipulations applied in (close surrounding of) target area such as pesticides, fungicides, fertilization, irrigation, and pruning
	Tolerance to high population density (e.g., intraspecific interactions), when released in high numbers	Tolerant to conspecific female parasitoids, ability to recognize already parasitized *D. suzukii* larvae/pupae, low migration rate in response to increasing parasitoid density
	Able to kill the pest and reduce pest population density within species community present in the target area, for example, by: Avoidance or be a strong competitor of predators and/or other species present in target area Being compatible with other natural enemies of the pest in such a way that they together result in higher killing efficiency	No/limited effect of presence of predators of parasitoids, such as hyperparasitoid *P. vindemmiae* or ants. Avoidance of multiparasitism or superior competitor during multiparasitism Preference for other life stages of the pest or microclimate than other natural enemies of *D. suzukii* present in target area
Environmental safety	No effect on abundance of other organisms in the ecosystem of release and notably in nontarget areas, either directly (e.g., killing nontarget herbivores or through intraguild predation) or indirectly (e.g., through competition for resources)	Relatively host specific, no hyperparasitoid to limit adverse effects on population density of other (beneficial) parasitoids and other *Drosophila* species present
	Low dispersal ability to limit negative effects in nontarget areas	Low dispersal tendency to other habitats (e.g., forests), low fly capacity, low passive dispersal (e.g., by air or human transport)
	No vector of (transferable) diseases/parasites which may affect wild strains or other species including humans, no effect on public health (e.g., toxic or allergic responses)	No carrier of Wolbachia strains that cause cytoplasmic incompatibility (CI) when outcrossing to wild strains
	Low chance of hybridization with closely related species in target area	Inability to mate and produce viable offspring with other parasitoid species present in area of release
	Inability to permanently establish outside release area to reduce risks in nontarget systems	High mortality rate in winter conditions in nontarget areas
Cost‐efficient (mass) rearing, stored, transport, and release	Maintenance of large population size for release, without inbreeding problems	High female fecundity, high survival rate, short developmental time, female‐biased sex ratio, high longevity
	Able to rear agent on target pest or closely related species that is relative cheap in production, without losing effectiveness against the target pest in area of release	Culture parasitoids on *D. suzukii* and/or other *Drosophila* species without losing effectiveness against *D. suzukii* pest. In case cultured on *D. suzukii*, able to separate parasitized and nonparasitized hosts before transport and release
	Able to rear agent that is efficient against all varieties of the target pest, to account for potential intraspecific differences between pest populations	Able to culture parasitoid that is efficient against different *D. suzukii* populations, for example, of different resistance levels
	Able to rear agent in conditions that enable efficient production (e.g., fast development, high density), without losing effectiveness in the field (e.g., by choosing conditions similar as target area such as temperature, photoperiod, and pest‐habitat stimuli)	Ability to learn host‐habitat cues (e.g., fruit color and odor) to increase pest‐killing efficiency, able to rear at relative high temperature enabling fast development time without loss of effectiveness upon release
	Long‐term storage (>weeks) with minimal fitness effects on, in particular, killing efficiency of the pest	Long‐term survival at, for example, low temperature (e.g., 10°C) as adult or immature stage, or by inducing diapause without loss of fitness (e.g., survival, fecundity, pest‐killing efficiency)
	Able to transport and release the agent to/in target area without negative effect on fitness	Survive transportation hazards, such as changes in temperature and mechanical impact of boxes being shaken. Possibility of using a banker system for parasitoid release, for example, artificial medium containing alternative hosts (nonpest), as well as parasitized larvae and pupa of different ages

*Note*

Performance is defined as the ability of an agent to suppress the pest population in the target area and to cost efficiently be (mass) reared and transported. Biocontrol traits that determine performance are composed of trait values across multiple species traits. Examples of important species trait values are listed for biocontrol agents in general as well as for parasitoids of *D. suzukii* specifically. Agents should preferably meet all four performance requirements. Note that trait values can differ depending on management goals (e.g., duration of effect in terms of number of generations or seasons).

**Figure 2:**
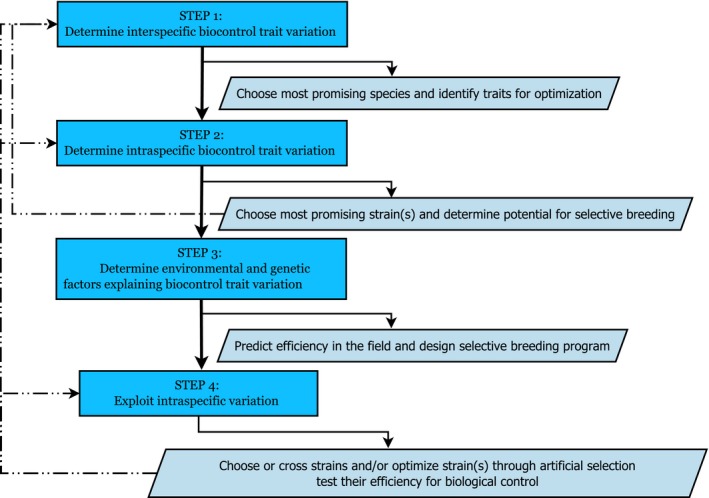
Proposed four‐step approach to exploit natural variation to optimize natural enemies as biological control agent. The approach involves exploitation of heritable as well as nonheritable variation. See text for detailed explanation of each step. Arrows on the left, after steps 2 and 4, refer to the case when the candidate control agent does not meet all requirements. In case the most promising species does not show intraspecific variation for the trait to be optimized (step 2), another species has to be chosen (step 1). In case the potential agent does not meet all requirements for biocontrol after testing their efficiency (step 4), further optimization is needed (step 4) or another species/strain should be chosen as potential biocontrol agent (steps 1 and 2)

Following the traditional method of biocontrol development, *the first step is to investigate the interspecific variation of natural enemies for relevant biocontrol traits,* to choose the most promising agent that best expresses all the required biocontrol characteristics (Figure 2, Table 1). The use of native natural enemies is preferred, and exotic species should only be used as second option to decrease biodiversity risks and circumvent the long process of obtaining importation and release permits. In the case of drosophilids, parasitoids are an important natural enemy that can cause high mortality in natural populations (Driessen, Hemerik, & Van Alphen, 1989; Fleury et al., 2004; Janssen, Driessen, De Haan, & Roodbol, 1987; Keebaugh & Schlenke, 2014). In addition, parasitoids are often effectively used as biocontrol agent due to their relative short generation time, ease to breed in the laboratory, and high host specificity and efficiency in killing the pest (MacQuarrie, Lyons, Seehausen, & Smith, 2016; Stiling & Cornelissen, 2005). Optimal biocontrol trait values for parasitoids of *D. suzukii* rely, for example, on host localization in ripening fruits, rather than the rotting fruits of the indigenous fruit‐breeding *Drosophila*, and high virulence to suppress the hosts’ immune system (Table 1).

When the selected species shows suboptimal performance for relevant biocontrol traits, they should be subject to optimization. So far, indigenous parasitoids that occur in the invaded area of *D. suzukii,* and that have been studied, have low killing efficiency against *D. suzukii* (Chabert et al., 2012; Kacsoh & Schlenke, 2012), which hinders their use as biocontrol agent. However, individuals of some parasitoid species are able to parasitize *D. suzukii* and cause fly death and/or can complete their development upon parasitizing the fly, indicating that there is potential/latent compatibility between these parasitoid species and the (new) host. Their killing efficiency should therefore be a main target for optimization.

To determine the potential for optimization of traits, knowledge of the extent and mechanistic basis of natural variation in the traits is required. Thus, *the second step is to investigate the intraspecific variation*. Phenotypic differences among strains of the same natural enemy species are a first indication that genetic trait variation may exist, which may be exploited for developing of a (more) effective biocontrol agent assuming that the variation is heritable. However, phenotypic variation might also be influenced by environmental factors (e.g., due to developmental stochasticity or the phenotype of the pest). This would limit the response to artificial selection as phenotypic variation can only be subject to selective breeding when it is (partly) heritable. In addition, the target area for biocontrol is an important aspect of the optimization as agents may perform better in a particular climate (e.g., Mediterranean vs. tropical climate) and/or existing insect communities (e.g., Europe vs. North America). Thus, we need to characterize the amount of phenotypic variation in the biocontrol traits that limit the effectiveness of the biocontrol agent (Box 1).

BOX 1 **Phenomics of biocontrol agents and pests**

1 Compared to the field of plant and livestock breeding, selective breeding of biological control agents is a relatively new field of study. Plant and animal breeding has been greatly advanced by new gene technologies: Most economically important plants and livestock have been sequenced (Edwards & Batley, 2010; Jackson, Iwata, Lee, Schmutz, & Shoemaker, 2011; Michael & Jackson, 2013), and this information can be used to improve and speed up breeding with techniques such as marker‐assisted selection and genomic selection. Linking phenotype and genotype however has become a bottleneck to further improve breeding success, as research on precise and efficient quantification of phenotypes has not kept pace with genomics (Furbank, 2009; Houle et al., 2010; Jackson et al., 2011; White et al., 2012). This holds in particular for complex traits that are controlled by multiple genes and subject to environmental influence. In plants, and to a limited extent in livestock, this has led to an emerging new field of investigation: phenomics, the large‐scale and systematic study of the phenome (all possible phenotypes). In particular, plant phenotypes can be measured at large scale with advanced nondestructive technologies, so‐called high‐throughput phenotyping (HTP), such as fluorescence imaging and near‐infrared reflectance spectroscopy to measure photosynthetic performance and composition of plant tissue (Araus & Cairns, 2014). Accurate and efficient measuring of phenotypes aids the understanding of underlying (genetic) mechanisms (reverse phenomics) and the screening of phenotypes to, for instance, choose the best strains for breeding (forward phenomics) (Furbank & Tester, 2011). History of animal and plant breeding underlines the importance to have insight into phenotypic variation to improve their performance for agriculture. It also stimulates (re)thinking about how biocontrol agents’ phenotypes can be systematically and accurately measured across time and space for improvement of biocontrol strategies.

Measuring and understanding phenotypic variation is of great importance for the development of biocontrol agents. In line with plant and livestock phenomics, biocontrol phenomics would entail the accurate and systematic (wide scale) phenotypic data collection of the candidate agent (species, strains or genotypes) and the target pest population(s) in relevant field and rearing conditions across time (e.g., through lifetime and season of agent and pest) and scale (all possible relevant habitats and thus biotic and abiotic conditions). This can aid solving major challenges in the development of control agents: (a) finding suitable agents, (b) predicting their success in a particular agricultural environment, (c) determining conditions for optimal performance, and (d) evaluating whether these conditions can be altered, and (e) identifying characteristics of important biocontrol trait values. In addition, it is also of importance for selective breeding to (f) set conditions for selective breeding, and (g) predict in which way and to what extent agents can be improved by artificial selection. The feasibility for large‐scale phenotyping is still limited, especially for arthropods, due to economical and practical (e.g., mobility) limitations and their low detectability in the field (small size). However, their relative short generation time and small size, compared to livestock, facilitate phenotyping in laboratory settings. Microbes are already being screened on large scale for their application as control agent (Figueroa‐Lopez, Cordero‐Ramirez, Quiroz‐Figueroa, & Maldonado‐Mendoza, 2014; van Lenteren et al., 2018; Stewart, Ohkura, & Mclean, 2010). To measure phenotypes of arthropod agents and their effect on the target pest population, tools such as sensors, imaging, and cameras, can be used to increase accuracy and scale to determine, for instance, stress response of pests in the presence of an agent and the presence, distribution and movement of the agents and the pests in the field and/or in the laboratory. These tools are already used in other fields of study (Nansen, Coelho, Vieira, & Parra, 2014; Nansen, Ribeiro, Dadour, & Roberts, 2015; Reynolds & Riley, 2002), although most seem to be especially feasible at only small scales. It would be interesting to make them applicable in the future at larger scales. Moreover, imaging technologies for plant phenomics such as the detection of plant health and plant responses to pests in the absence and presence of biocontrol agents (Abdel‐Rahman et al., 2017; Reynolds & Riley, 2002; Wang, Nakano, Ohashi, Takizawa, & He, 2010; Zhou, Zang, Yan, & Luo, 2014) can also be used to measure success of biocontrol. The difficulty is that the success of a control agent does not only depend on genotype × environment interactions as most target traits in animal and plant breeding (except for pest resistance) but on an even more complex two‐species × environment interaction (Figure 1). The four‐step approach proposed in this review displays how phenomics can be applied to biocontrol. The first and second steps (investigation of inter‐ and intraspecific variation) are analogous to forward phenomics, that is, screen and choose natural enemies with desired phenotypes for biocontrol traits. The third step (investigation of factors that shape the variation) can be seen as reverse phenomics, to discover mechanisms of variation and which helps to set the conditions for optimal trait expression.

In which way and to what extent intraspecific variation can be exploited for optimization depends on the genetic basis of, and (stochastic) environmental effects on, the expression of the trait of interest. Hence, *the third step is to determine environmental and genetic factors that shape the biocontrol trait variation*. Insight into the amount of genetic variation and genetic architecture of traits may aid the design of a breeding plan and prediction of the response to selection, as well as anticipate potential trade‐offs and genetic correlated responses (Lommen et al., 2017). Selection on a target trait can change the investment in (trade‐off) or the expression of another trait (correlated response), resulting in an unintentional change in a nontarget trait. This does not always have to be negative; the effect might also be exploited during selection. Note that biocontrol traits are composite traits (Table 1). Trade‐offs and correlated responses might therefore either (a) occur between traits determining the same biocontrol trait like “killing efficiency” (such as attack rate and host immune suppression ability) or (b) between traits determining two different biocontrol traits such as “killing efficiency” and “robustness under (a)biotic conditions” (e.g., between killing efficiency and survival rate). In addition, environmental factors can also influence trait value expression (Figure 1). However, the pest and natural enemy may be affected differently by the same environmental factors, which may have an impact on their interaction. Therefore, identification of genetic and environmental factors affecting the target trait of the candidate agent is required to predict its field efficiency and to set optimal breeding conditions to secure its success in the field.

Measuring phenotypic variation (of the control agent) in a relevant range of (agricultural and rearing) conditions can give insight into the extent of phenotypic plasticity (i.e., the different phenotypes a genotype can produce in different environments), and which environmental factors influence expression of the trait(s) of interest. The collection of all possible phenotypes across time (e.g., developmental stages) and space (e.g., geographic regions) is called the “phenome” (Houle, Govindaraju, & Omholt, 2010; Soule, 1967) (see also Box 1). This knowledge can be used to identify environmental factors that may constrain the performance of an agent. Moreover, it can yield insights into trade‐offs that may hamper the adaptive response and thus to (a) predict the success of artificial selection and (b) design a breeding program (Figure 2, step 3). In addition, agents will encounter different and a greater number of variable biotic and abiotic factors in the field than under laboratory conditions. This may influence their killing ability of the pest. For example, temperature differences and the presence of competitors can alter the agents’ performance in the field (Andrade, Pratissoli, Dalvi, Desneux, and Santos Junior (2011); Boivin and Brodeur (2006). Hence, knowledge about environmental effects is also required to (c) predict the performance of the agent in the field. In an interesting manner, insight into sources of variation can also be used to (d) identify additional methods to optimize performance of the agent, by exploitation of nonheritable variation, for example, by learning ability of the agent or alteration of environmental conditions in the greenhouse to increase killing efficiency of the pest.

At last, *the fourth step is to exploit the available variation and select (for) an agent with the most optimal combination of phenotypic traits*. This can be either through (a) choosing the most competent strain for the target area (“strain selection”), (b) crossing populations present in the invaded area and/or with ones that are native of the pest (“cross‐breeding”), and/or (c) optimization of a genetically variable strain through artificial selection (“selective breeding”). The optimization approach can be applied iteratively, each time identifying the limiting factors for the effectiveness of the biocontrol agent, and selecting on (trait values of the) different biocontrol traits. At each round, the selected agent should be tested for its ability to be mass reared and for its performance success in the target area, to assess whether it can be implemented in pest management, whether it needs further improvement, or whether another candidate agent has to be selected in case it shows no potential (Figure 2).

Below, we review current knowledge of *D. suzukii*–parasitoid interactions in more detail following our proposed four‐step approach and point at ways to optimize parasitoids from the invasive area to develop efficient biological control agents.

## 3 STEPS 1 AND 2: EXPLORING INTER‐ AND INTRASPECIFIC VARIATION IN KILLING EFFICIENCY

### 3.1 Parasitoids in the invasive area: Europe and North America

Several surveys performed in Europe (France, Spain, Italy, and Switzerland) and North America (Canada, USA, and Mexico) explored the ability of native parasitoids to parasitize the invasive *D. suzukii*. A total of 17 parasitoid species have been investigated.

#### 3.1.1 Interspecific variation

In only 24% of the investigated species, a population has been found with a high parasitization success rate (61%–100%, Table 2). Two pupal parasitoids, *Pachycrepoideus vindemmiae* and *Trichopria Drosophilae,* were repeatedly reported to parasitize and emerge from *D. suzukii*. Two other pupal parasitoids, *Spalangia erythromera* and *Vrestovia fidenas,* and one larval parasitoid, *Leptopilina heterotoma*, were recorded once (Table 2). Other species, in particular those that parasitize the larval stage, such as *Asobara tabida*,* Leptopilina clavipes,* and *Leptopilina boulardi,* did not survive in or emerge from *D. suzukii* (Table 2). Thus, there is clear interspecific variation between parasitoids in their success to parasitize *D. suzukii*, and most indigenous parasitoid species that have been studied are unable to complete their development on *D. suzukii* hosts.

**Table 2:** Overview of parasitoids occurring in the newly invaded area (mostly Europe and North America), investigated for their ability to parasitize *Drosophila suzukii* in the field and/or the laboratory

Natural enemy	Country/state	Documented parasitoids of *D. suzukii* in the field	Parasitization success in the laboratory and encapsulation rate	Fly infestation rate (infestation) or coupled fly and parasitoid death (inadequacy)	Reference
Pupal parasitoids					
*Pachycrepoideus vindemmiae*	Mexico	Yes, on infested *D. suzukii* traps			Cancino et al. (2015)
	France		Serrières population: yes, medium success	High infestation	Chabert et al. (2012)
			Maison Neuve population: medium success (populations do not differ sig.)	Medium infestation	
	Spain	Yes, on infested *D. suzukii* traps	Yes, high success	High infestation	Gabarra et al. (2015)
	Switzerland		Yes, high success		Knoll et al. (2017)
	Italy	Yes, on infested *D. suzukii* traps	Yes, medium success	No inadequacy	Stacconi et al. (2013)
		Yes, on infested *D. suzukii* traps (mean: 0.35 parasitoid/trap)			Miller et al. (2015)
			Yes, medium success	Medium infestation	Stacconi et al. (2015)
	California	Yes, on field‐collected fruits (unpublished data)	Yes, successful	Fruits: medium–high infestation; soil: low–medium infestation (fruit vs. soil differ sig.)	Wang et al. (2016b)
	Oregon	Yes, on infested *D. suzukii* traps			Stacconi et al. (2013)
		Yes, on infested *D. suzukii* traps (mean: 1.93%–6.06 parasitoids/trap)			Miller et al. (2015)
			First‐instar and second‐instar larvae: no success Third‐instar pupae: yes, medium–high success	First‐instar and second‐instar larvae: low infestation Third‐instar pupae: high infestation	Stacconi et al. (2015)
*Pachycrepoideus* sp.	Georgia		Yes, low success	Low inadequacy	Kacsoh and Schlenke (2012)
*Trichopria. cf. Drosophilae*	Mexico	Yes, on infested *D. suzukii* traps			Cancino et al. (2015)
France		Ste Foy population: yes, low success	SF population: high infestation	Chabert et al. (2012)
		Sablons population: yes, high success (populations differ sig.)	SA population: high infestation (SF and SA populations differ sig.)	
Spain	Yes, on infested *D. suzukii* traps and field‐collected fruits (parasitization rate fruits 3.8%–10.7%)	Yes, high success	Medium infestation	Gabarra et al. (2015)
California	Yes, on field‐collected fruits (unpublished data)	Yes successful	Medium–high infestation	Wang et al. (2016b)
Switzerland		Vaud strain: yes, high success		Knoll et al. (2017)
		Ticino strain: yes, medium success (populations differ sig.)		
Italy		Yes, high success	No inadequacy	Mazzetto et al. (2016)
		Yes, high success		Stacconi et al. (2015)
*Trichopria sp*.	California		Yes, high success	Low inadequacy	Kacsoh and Schlenke (2012)
	France		Yes, high success	No inadequacy	Kacsoh and Schlenke (2012)
*Spalangia simplex*	Mexico	Yes, on infested *D. suzukii* traps			Cancino et al. (2015)
*Spalangia erythromera*	Switzerland		Yes, high success		Knoll et al. (2017)
*Vrestovia fidenas*	Switzerland		Yes, low success		Knoll et al. (2017)
Larval parasitoids					
*Asobara tabida*	France		Igé population: no success (oviposit in 1.25% larvae).		Chabert et al. (2012)
			Sablons population: no success		
			No success. high encapsulation rate	Low inadequacy	Kacsoh and Schlenke (2012)
	Sweden		No success. medium encapsulation rate	Low inadequacy	Kacsoh and Schlenke (2012)
	Switzerland		No success	No inadequacy	Knoll et al. (2017)
*Asoara citri*	Ivory Coast		Yes, very low success. Low encapsulation rate	High inadequacy	Kacsoh and Schlenke (2012)
*Aphaereta sp*.	Georgia		No success, medium encapsulation rate	Very low inadequacy	Kacsoh and Schlenke (2012)
*Leptopilina clavipes*	Netherlands		No, high encapsulation rate	Medium inadequacy	Kacsoh and Schlenke (2012)
*Leptopilina heterotoma*	France		St Etienne/Chalaronne population: no success, high encapsulation rate	Medium infestation	Chabert et al. (2012)
	Antibes population: very low success, high encapsulation rate	High infestation (ST and AN populations differ significantly in infestation)			
	French *D. suzukii* strain: no success, high encapsulation rate	Low inadequacy	Poyet et al. (2013)
	Japanese *D. suzukii* strain: no success, medium–high encapsulation rate	Medium inadequacy	
Oregon	Yes, on infested *D. suzukii* traps (mean: 0–0.06 parasitoid/trap)			Miller et al. (2015)
		No success		Stacconi et al. (2015)
Italy	Yes, on infested *D.suzukii* traps (mean: 1.01 parasitoid/trap)			Miller et al. (2015)
		No success	Medium adequacy	Mazzetto et al. (2016)
		Yes, low.–medium encapsulation rate	Medium–high infestation	Stacconi et al. (2015)
California		No success, high encapsulation rate	Medium inadequacy	Kacsoh and Schlenke (2012)
		No success		Stacconi et al. (2015)
Sweden		No success, high encapsulation rate	Low inadequacy	Kacsoh and Schlenke (2012)
Switzerland		Yes, very low success	Low (average) inadequacy, significant differences between strains	Knoll et al. (2017)
*Leptopilina victoriae*	Hawaii		No success, high encapsulation rate	Medium inadequacy	Kacsoh and Schlenke (2012)
*Leptopilina boulardi*	Mexico	Yes, on infested *D. suzukii* traps			Cancino et al. (2015)
France		Sablons population: no success, medium encapsulation rate	Medium infestation	Chabert et al. (2012)
		Eyguières population: no success, medium encapsulation rate (populations do not differ sig.)	High infestation	
		No success, high encapsulation rate	Low inadequacy	Kacsoh and Schlenke (2012)
Italy		No success	No inadequacy	Mazzetto et al. (2016)
Congo		No success, high encapsulation rate	Medium inadequacy	Kacsoh and Schlenke (2012)
Kenya		No success, high encapsulation rate	Medium inadequacy	Kacsoh and Schlenke (2012)
California		No success, high encapsulation rate	Medium inadequacy	Kacsoh and Schlenke (2012)
Switzerland		No success	Low inadequacy	Knoll et al. (2017)
*Leptopilina guineaensis*	Cameroon		Yes, low success. High encapsulation rate	Medium inadequacy	Kacsoh and Schlenke (2012)
	South Africa		No success, medium encapsulation rate	Medium inadequacy	Kacsoh and Schlenke (2012)
*Ganaspis xanthopoda* a	Hawaii		Yes, very low success. High encapsulation rate	Low inadequacy	Kacsoh and Schlenke (2012)
	Uganda		No success, high encapsulation rate	Low inadequacy	Kacsoh and Schlenke (2012)
*Ganaspis* sp.	Florida		Yes, low success. High encapsulation rate	High inadequacy	Kacsoh and Schlenke (2012)
	Hawaii		Yes, medium success. High encapsulation rate	Medium inadequacy	Kacsoh and Schlenke (2012)

*Notes*

Field surveys include the placement of traps (*D. suzukii‐*infested or *D. suzukii*‐uninfested fruit‐baited traps), and/or the collection of fruits from natural habitats or crops. Laboratory essays were performed to test the ability of parasitoids to parasitize *D. suzukii* by exposure of larvae/pupae to the parasitoid(s) in a no‐choice test. Parasitization success (rate) is the percentage of parasitoids that eclosed from *D. suzukii*. Due to variable experimental setup and calculations, parasitization success rate is categorized in “no” (no parasitoid emergence), “very low” (<10% success rate), “low” (10%–29%), “medium” (30%–60%), and “high” (61%–100%). When examined, fly infestation rate (infestation) or coupled fly and parasitoid death (inadequacy) are presented. Fly infestation rate includes fly death due to parasitoid emergence and/or coupled fly and parasitoid death (inadequacy). Note that comparing the parasitization results of these studies, in particular quantitative outcomes, is complicated as different calculations and experimental methods were used. In addition, host genetic backgrounds may differ between studies and influence results. Therefore, the reported parasitization rates should be interpreted cautiously for their extrapolation to real‐world applications.

a Reported as *G. xanthopoda,* but would be *G. brasiliens* as described by Nomano et al. (2017).

#### 3.1.2 Intraspecific variation

Although most parasitoid species could not successfully parasitize *D. suzukii*, intraspecific variation indicates potential future adaptation to the pest. For example, French *A. tabida* strains collected from Igé and Sablons showed little to no attempt (0%–1.25%) to oviposit in *D. suzukii* larvae (Chabert et al., 2012), whereas a Swedish strain and another French strain collected in Sospel showed an infestation rate of about 50% and 80%, respectively (Kacsoh & Schlenke, 2012). Also, whereas *L. boulardi* was not able to emerge from *D. suzukii*, Chabert et al. (2012) reported that they do oviposit in *D. suzukii* and induce high host mortality. Between‐population differences in parasitization success were also found among the three species capable of successfully parasitizing *D. suzukii* (Table 2). *Leptopilina heterotoma* from Oregon, northwest Italy, France, California, Sweden, and Switzerland were not able to complete their life cycle when parasitizing *D. suzukii* in the laboratory (Chabert et al., 2012; Kacsoh & Schlenke, 2012; Knoll, Ellenbroek, Romeis, & Collatz, 2017; Mazzetto et al., 2016; Poyet et al., 2013; Stacconi et al., 2015), but an Italian population from Trento could (Stacconi et al., 2015). Furthermore, wasps from a French population were not able to overcome the flies’ immune defense to produce viable offspring, although, similar to another population from North Italy (Lombardy and Piedmont), they did oviposit and caused fly death (Chabert et al., 2012; Mazzetto et al., 2016). In an interesting manner, when *D. suzukii* larvae were parasitized by four individuals, rather than a single wasp, some parasitoids developed and eclosed (Chabert et al., 2012). Populations of parasitoid *T. drosophilae* also differed in their performance on *D. suzukii. *For example, the success rate differed between two populations within France (Chabert et al., 2012), and between populations from South Korea and California in which the Californian population unexpectedly performed significantly better on *D. suzukii* than the Korean population (Wang, Kacar, Biondi, & Daane, 2016b). These cases provide clear evidence for the existence of intraspecific variation in parasitization ability between populations of known indigenous *D. suzukii* parasitoids.

### 3.2 Parasitoids in the native area: Asia

The parasitoid species that attack *D. suzukii* populations in the area of origin, Asia, have not been thoroughly investigated. The first publications on natural enemies of *D. suzukii* only appeared in 2007 (Mitsui, van Achterberg, Nordlander, & Kimura, 2007), and research has mainly focused on parasitoid species in Japan and to a limited extent on species from China and Korea (Table 3). A total of two pupal and 14 larval parasitoids have been identified that are able to parasitize *D. suzukii* (Table 3). Most of them belong to *Asobara, Ganaspis,* or *Leptopilina*, but these parasitoids also show differences in parasitization success.

**Table 3:** Overview of parasitoids from Asia investigated for their ability to parasitize *D. suzukii* in the field and/or in the laboratory

Natural enemy	Country	Documented parasitoids of *D. suzukii* in the field	Parasitization success in the laboratory (rate given when possible)	Reference
Pupal parasitoids				
*Trichopria Drosophilae*	Korea	Yes, on uninfested traps	Yes	Daane et al. (2016)
	China	Yes, on infested *D. suzukii* traps		Zhu, Li, Wang, Zhang, and Hu (2017)
*Pachycrepoideus vindemmiae*	Korea	No, only on other drosophilids	Yes	Daane et al. (2016)
Larval parasitoids				
*Asobara species* (unidentified)	Japan	Yes, on field‐collected fruits. <1%^a^		Kasuya et al. (2013)
*Asobara japonica*	Japan	Yes, on uninfested traps. 0.2% parasitism rate		Mitsui et al. (2007)
			Yes, high	Mitsui and Kimura (2010)
		No, only from other drosophilids	Yes, medium	Ideo et al. (2008)
		Yes, on field‐collected fruits. 0.2% parasitism rate^a^		Nomano et al. (2015)
			Yes, high	Kacsoh and Schlenke (2012)
			Yes, medium (21°C) to high (°25C)	Chabert et al. (2012)
	Korea	Yes, on infested *D. suzukii* traps		Guerrieri et al. (2016)
		Yes, on uninfested traps and field‐collected fruits	Yes	Daane et al. (2016)
*Asobara leveri*	Korea	Yes, on infested *D. suzukii* traps		Guerrieri et al. (2016)
	Korea	Yes, on uninfested traps and field‐collected fruits		Daane et al. (2016)
*Asobara brevicauda*	Korea	Yes, on field‐collected fruits		Daane et al. (2016)
*Asobara tabida*	Japan	Yes, on uninfested traps. 0.1% parasitism rate		Mitsui et al. (2007)
		Yes, on field‐collected fruits. 0.2% parasitism rate	No, but oviposition observed	Nomano et al. (2015)
*Asobara rossica*	Japan	Yes, on field‐collected fruits. About 0.05%^a^ parasitism rate	No, but oviposition observed	Nomano et al. (2015)
*Asobara rufescens*	Japan	Yes, on field‐collected fruits. About 0.05%^a^ parasitism rate	No, but oviposition observed	Nomano et al. (2015)
*Asobara pleuralis*	Japan		No	Nomano et al. (2015)
	Indonesia		No success. high encapsulation rate	Kacsoh and Schlenke (2012)
*Asobara* sp. TS1^b^	Japan	Yes, on field‐collected fruits. 4.8%^a^ parasitism rate	Yes, low	Nomano et al. (2015)
*Ganaspis brasiliensis*	Japan	Yes, on uninfested traps. 3.9% parasitism rate (“*D. suzukii*‐type”)^c^		Mitsui et al. (2007)
			No. very low infestation rate (3.3% parasitized) (“*D. lutescenes* type”)^c^	Mitsui and Kimura (2010)
		Yes, on field‐collected fruits. 4%–7% parasitism rate (“*D. suzukii*‐type”)^c^	Yes, low (only from fruits, but not from artificial diet) (“*D. suzukii‐*type”)^c^	Kasuya et al. (2013)
		Yes, on field‐collected fruits. (“*D. suzukii*‐type”) ^c^		Nomano et al. (2015)
	Korea	Yes, on field‐collected fruits	Yes	Daane et al. (2016)
*Leptopilina japonica japonica*	Japan	Yes, on field‐collected fruits. <1%^a^ parasitism rate		Kasuya et al. (2013)
	Korea	Yes, on field‐collected fruits	Yes	Daane et al. (2016)
*Leptopilina japonica formosana*	Korea	Yes, on field‐collected fruits		Daane et al. (2016)
*Leptopilina boulardi*	Korea	No, only from other drosophilids		Daane et al. (2016)
*Leptopilina japonica victoriae*	Philippines		No success, medium 50% encapsulation rate	Kacsoh and Schlenke (2012)

*Notes*

Field surveys include the placement of traps (*D. suzukii‐*infested or *D. suzukii*‐uninfested fruit‐baited traps), and/or the collection of fruits from wild habitats or crops. Laboratory essays were performed to test the ability of parasitoids to parasitize *D. suzukii* by exposure of larvae/pupae to the parasitoid(s) in a no‐choice test. Parasitization success (rate) is the percentage of parasitoids that eclosed from *D. suzukii*. Due to variable experimental setup and calculations, parasitization success rate is categorized in “no” (no parasitoid emergence), “very low” (<10% success rate), “low” (10%–29%), “medium” (30%–60%), and “high” (61%–100%). When parasitism rate was not calculated in the study, estimations were made by dividing number of emerged parasitoids by total number of presented/collected flies when possible. These estimations are indicated by the symbol “a”. Note that comparing the parasitization results of these studies, in particular quantitative outcomes, is complicated as different calculations and experimental methods were used. In addition, host genetic backgrounds may differ between studies and influence results. Therefore, the rates that have been reported here should be interpreted cautiously for their extrapolation to real‐world applications.

Undescribed species from Japan. ^c^Previously assigned as *G. xanthopoda,* but later identified as *G. brasiliens* by Nomano et al. (2017)*. *There seem to be different types: one specialized on *D. suzukii* (“*D. suzukii*‐associated type”) and one unable to parasitize *D. suzukii* and mainly parasitize *D. lutescens* (“*D. lutescens‐*associated type”) (Kasuya et al., 2013; Nomano et al. 2017).

#### 3.2.1 Interspecific variation

Of the 16 investigated parasitoid species, 88% are able to successfully parasitize *D. suzukii* in the field and/or in the laboratory*. *Only *A. pleuralis* and *L. boulardi* were not observed to emerge from *D. suzukii* at all (Daane et al., 2016; Nomano et al., 2015). The large variation in parasitization behavior can be illustrated with the *Asobara* genus. There are large differences among species within this genus in their ability to accept *D. suzukii* for oviposition and successful development to adulthood*:* While *A. pleuralis* did not oviposit in *D. suzukii* (Nomano et al., 2015), *A. tabida, A. rufescens,* and *A. rossica* did oviposit but all individuals died in the fly host (Nomano et al., 2015). Only *A. *sp. TS1, *A. *sp. TK1, *A. japonica, A. leveri,* and *A. brevicauda* would readily accept *D. suzukii* for ovipositon and were able to complete development (Daane et al., 2016; Guerrieri, Giorgini, Cascone, Carpenito, & van Achterberg, 2016; Ideo, Watada, Mitsui, & Kimura, 2008; Kacsoh & Schlenke, 2012; Mitsui & Kimura, 2010; Nomano et al., 2015). In an interesting manner, while *A. tabida, A. rufescens, and A. rossica* could not complete their development while parasitizing *D. suzukii* in the laboratory, they emerged from flies collected in the field, indicating that these parasitoids can survive on this host (Nomano et al., 2015).

#### 3.2.2 Intraspecific variation

Parasitization success varies between and within populations of the same species. The *Asobara. *sp. TS1 population of Tsushima (Japan), for example, is able to develop in *D. suzukii,* although individuals differed in success: 83.3% died in the larval stage and only 13.3% of the individuals were able to complete development and eclose (Nomano et al., 2015). An interesting example of between‐population differences is the parasitoid *Ganaspis brasiliensis,* of which there are different “types” that differ in host use, morphology, nucleotide sequence, and geographic distribution (Kasuya, Mitsui, Ideo, Watada, & Kimura, 2013; Nomano et al., 2017). One has *D. lutescenes* as its main host and has limited success when parasitizing *D. suzukii*, the other is specialized on *D. suzukii* and can successfully parasitize *D. suzukii* but not *D. lutescenes* (Kasuya et al., 2013). In addition, differences in parasitization success between populations have been found for *A. japonica* collected in the surroundings of Tokyo: One study recorded 80% eclosion of the parasitoid (Kacsoh & Schlenke, 2012), another study an eclosion rate of only 44% (Ideo et al., 2008), and Mitsui and Kimura (2010) found an eclosion success of 67%, suggesting there is substantial variation between parasitoid populations.

## 4 STEP 3: UNDERSTANDING VARIATION IN D. SUZUKII–PARASITOID INTERACTION

The killing efficiency of parasitoids depends on a complex two‐species interaction (Figure 1). Below, we review what has been investigated as causal mechanisms for the phenotypic variation, and the environmental and genetic factors that can shape the interaction and coevolution of *D. suzukii* and their parasitoids. Moreover, we describe how these factors can aid the development of biological control agents.

### 4.1 Sources of variation in *D. suzukii*

#### 4.1.1 Phenotypic variation and its causal mechanisms

The resistance level of the host is an important trait determining the outcome of host–parasitoid interactions. Like several other *Drosophila* species, *D. suzukii* can protect itself from parasitoids by melanotic encapsulation of the wasps’ egg (Chabert et al., 2012; Kacsoh & Schlenke, 2012). Its immune response, however, seems to be much stronger than *D. melanogaster* and most other drosophilids. This is attributed to the relatively high hemocyte count of *D. suzukii* (Kacsoh & Schlenke, 2012; Poyet et al., 2013), which enables it to mount a highly successful immune response toward a wide range of parasitoid species (Kacsoh & Schlenke, 2012).

#### 4.1.2 Genetic effects

The genetic basis and genetic variation of parasitoid resistance in *D. suzukii* have not yet been investigated. As genetic variation in resistance is reported for other *Drosophila* species (e.g., Dubuffet et al., 2007; Gerritsma, de Haan, van de Zande, & Wertheim, 2013; Kraaijeveld & Godfray, 1997), it is also likely to exist for *D. suzukii*. The amount of genetic variation in invasive species populations however depends on the size of the founder population, and the number and sources of additional introductions. When previously isolated populations start interbreeding (admixture events), the recombining of allelic variations can lead to increased genetic diversity. Throughout the course of the invasion of *D. suzukii*, its genetic diversity changed through bottlenecks and admixture events (Fraimout et al., 2017). A comparison of the host genotype across neutral markers (6–28 microsatellites) and six X‐linked loci in coding and noncoding sequences indicated relatively high intraspecific genetic variation within and between populations in the invaded regions (Adrion et al., 2014; Bahder, Bahder, Hamby, Walsh, & Zalom, 2015; Fraimout et al., 2015, 2017). It is therefore reasonable to assume that there is substantial intraspecific genotypic variation in the invaded populations that can contribute to the variable *D. suzukii*–parasitoid outcome.

#### 4.1.3 Environmental effects

Differences in biotic and abiotic environmental conditions can influence host resistance levels. By laying eggs in fruits rich in atropine, an entomotoxic alkaloid present in plants of the Solanaceae family*, D. suzukii* can enhance resistance to parasitoids via transgenerational medication (Poyet et al., 2017). Other abiotic factors that affect the immune response in drosophilids are temperature (Fellowes, Kraaijeveld, & Godfray, 1999; Fleury et al., 2004), and host diet (Anagnostou, LeGrand, & Rohlfs, 2010; Ayres & Schneider, 2009; Howick & Lazzaro, 2014; Meshrif, Rohlfs, & Roeder, 2016). In addition, an important biotic factor affecting the immune response is microbes. In *Drosophila,* the microbiome can affect immunity by increasing (Teixeira, Ferreira, & Ashburner, 2008; Xie, Butler, Sanchez, & Mateos, 2014) or decreasing resistance (Fytrou, Schofield, Kraaijeveld, & Hubbard, 2006), depending on microbial composition and/or host genetic background (Chaplinska, Gerritsma, Dini‐Andreote, Salles, & Wertheim, 2016). By experimental selection, it is possible to increase the ability of parasitoids to overcome the symbiont‐mediated resistance of the host (Rouchet & Vorburger, 2014). In an interesting manner, *D. suzukii* populations in the invaded area harbor the endosymbiont *Wolbachia pipientis* (“wSuz” strain) (Cattel, Martinez, Jiggins, Mouton, & Gibert, 2016; Cattel, Kaur, et al., 2016; Hamm et al., 2014; Mazzetto, Gonella, & Alma, 2015; Siozios et al., 2013; Tochen et al., 2014), a bacterium present in a wide range of arthropods that can manipulate the host's biology in different ways (see, e.g., Werren, Baldo, & Clark, 2008). In case of *D. suzukii,* it can mediate resistance toward RNA viruses (Cattel, Martinez, et al., 2016) and can increase female fecundity (Mazzetto et al., 2015). However, note that fitness effects might be depended on the wSuz variant, due to intra‐wSuz strain variation (Kaur, Siozios, Miller, & Rota‐Stabelli, 2017). It would be worthwhile to further investigate the role of *Wolbachia* and other microbes in the *D. suzukii*–parasitoid interaction.

#### 4.1.4 Implications for selection or selective breeding of a biocontrol agent

To assure high parasitization success of the control agent, a *D. suzukii* population has to be chosen for selective breeding (and later for mass rearing) similar to those in the target area. It is important to prime the agent for an efficient attack because there might be natural intraspecific variation in the level of resistance in *D. suzukii* in the invasive areas. The French *D. suzukii* strain has an hemocyte load that is about twice as high as the Japanese strains, and a higher encapsulation and parasitoid‐killing ability (Poyet et al., 2013). This suggests that the founding populations in Europe had a high immune response toward parasitoids and/or underwent a fast‐evolutionary change in resistance ability. Hence, to select and breed a control agent on a *D. suzukii* population, its level of resistance should be similar to the population in the target area. Therefore, more research is needed to investigate the amount of genetic variation in resistance in the invasive area. Moreover, knowledge of environmental conditions that are difficult to control, such as presence of atropine producing plants, may be of great importance to predict the success of the control agent.

To increase the success of a control agent, some factors that weaken the pest may be manipulated for pest management. The maintenance of the immune system in the absence of infection, and the investment in mounting a defense when infected, both have clear fitness costs, as resources allocated toward the immune system cannot be invested in other life history traits. *Drosophila melanogaster* for instance had a lower reproductive success after an immune challenge (Nystrand & Dowling, 2014) and lines selected for increased immunity had a lower larval competitive ability (Kraaijeveld & Godfray, 1997). Resource allocation can be influenced by environmental conditions. In stressful conditions, like insecticide exposure (Delpuech, Frey, & Carton, 1996), or high population density (Wajnberg, Prevost, & Boulétreau, 1985), resistance of *D. melanogaster* decreases. Intraspecific variation in *D. suzukii* defense can therefore occur due to differences in resource allocation. The energy balance of the pest can be exploited during pest management by, for example, stressing *D. suzukii* by combining control practices (e.g., a second biocontrol agent) or exposure to unfavorable climatic conditions, to make them more susceptible to parasitoids. Temperature outside the optimum range (±22–26°C) and low relative humidity (<71% RH) decrease the intrinsic rate of population increase of *D. suzukii* (Tochen et al., 2014, 2016). It would be interesting to investigate whether these factors also increase their susceptibility to parasitoids.

### 4.2 Sources of variation in parasitoids of *D. suzukii*

#### 4.2.1 Phenotypic variation and its causal mechanisms

Natural enemies require virulence strategies to overcome host resistance of *D. suzukii*. Most parasitoids in the invasive area, such as larval parasitoids *A. tabida*,* L. boulardi*,* L. victoriae,* and *G. xanthopoda,* do oviposit in *D. suzukii*, but their success rate is rather low as their mortality is nearly 100% (Table 2). The medium‐to‐high (30%–100%) ability of the generalist pupal parasitoids *P. vindemmiae* and *T.cf. drosophilae* to parasitize *D. suzukii* (Table 2) suggests a different parasitization strategy. As both species paralyze the host by injection of venom (Wang, Kacar, Biondi, & Daane, 2016a; Wang & Messing, 2004b) and pupae have compared to larvae no/limited resistance against parasitoids, these species have developed a highly virulent strategy that is nonspecies specific. The larval parasitoid *L. heterotoma* is also able to some (low) extent to successfully parasitize *D. suzukii*, or it can induce high fly mortality (Table 2). Along with the egg, *Leptopilina* injects virulence particles that modify host physiology, facilitating parasitization (Lee et al., 2009). The composition of these particles and their effect on the host differ between species and strains (Dupas, Brehelin, Frey, & Carton, 1996; Lee et al., 2009; Mortimer, 2013; Poirié, Carton, & Dubuffet, 2009), which therefore might play a role in the observed intraspecific variation in *D. suzukii*–parasitoid outcome.

Parasitization ability is also influenced by the parasitoid's ability to find the host. This depends on their ability to use host cues (e.g., pheromones, substrate odor and host tracks) (Dicke, Lenteren, Boskamp, & Voorst, 1985; Perez‐Maluf, Rafalimanana, Campan, Fleury, & Kaiser, 2008; Wertheim, Vet, & Dicke, 2003) and their experience with the host (habitat) (associative learning) (Kaiser, Perez‐Maluf, Sandoz, & Pham‐Delegue, 2003; Oliai & King, 2000; Papaj & Vet, 1990; Segura, Viscarret, Paladino, Ovruski, & Cladera, 2007). In the case of *D. suzukii,* host‐finding may be a challenge for the parasitoid, as (a) its main patch location (ripening fruits) is distinct from most other *Drosophila* species (Atallah, Teixeira, Salazar, Zaragoza, & Kopp, 2014; Atkinson & Shorrocks, 1977; Markow & O'Grady, 2008). Moreover, (b) its eggs are highly scattered (Mitsui, Takahashi, & Kimura, 2006; Poyet et al., 2014, 2015), which might make alternative highly infested patches of other drosophilids species more attractive and time‐efficient to exploit. Furthermore, due to its (c) recent invasion and (d) high immune response, parasitoids may not be able (yet) to recognize and successfully parasitize *D. suzukii*. These factors highlight the difference between laboratory and field experiments: Parasitoids able to successfully parasitize *D. suzukii* in the laboratory might not be able to localize the pest in the field. Parasitoids however have been found emerging from *D. suzukii* baited field traps in Europe and North America (Table 2) (Stacconi et al., 2013; Gabarra, Riudavets, Rodriguez, Pujade‐Villar, & Arno, 2015; Miller et al., 2015; A. Kruitwagen, unpublished results). However, due to limitations in experimental setups, no clear conclusions can yet be drawn on natural parasitization rates of *D. suzukii* relative to other drosophilids and on the parasitoid's ability and efficiency to localize and exploit *D. suzukii* host patches. Field experiments either only included *D. suzukii* baited traps (Gabarra et al., 2015), so parasitization could not be compared with other fruit flies, or baits were placed in such a way that parasitoids may be attracted to their co‐occurring natural *D. melanogaster* host (Miller et al., 2015), and/or the unnaturally high number of immature fruit flies in the baits (Miller et al., 2015; Stacconi et al., 2013). Hence, more research is needed to obtain insight into *D. suzukii*–parasitoid interaction in nature and to assess which factors might stimulate host‐finding ability.

#### 4.2.2 Genetic effects

Virulence, the ability to infest or harm the host, is determined at least partly by the genotype of the parasitoid (Carton & Nappi, 1989; Dubuffet et al., 2007; Dupas & Boscaro, 1999; Dupas, Frey, & Carton, 1998; Goecks et al., 2013; Kraaijeveld, Hutcheson, Limentani, & Godfray, 2001). A well‐studied example is the parasitoid *L. boulardi*, which shows intraspecific variation in its ability to suppress the host immune response in *D. melanogaster* and *D. yakuba* (Dubuffet et al., 2007; Dupas et al., 1998). Its virulence is determined by two immune suppressive genes encoded at different unlinked loci (Dupas & Carton, 1999). Two strains have been described with different genotypes, one that can successfully parasitize *D. melanogaster*, but not *D. yakuba*, and is homozygous for alleles for virulence against *D. melanogaster* but not against *D. yakuba* (Dubuffet et al., 2007). The other strain is homozygous for alleles for virulence against *D. yakuba* but not *D. melanogaster* (Dubuffet et al., 2007). In an interesting manner, contrary to what would be expected based on its genotype, this strain can also reproduce on *D. melanogaster. *This suggests that other factors, for example, *Drosophila* host genotype*,* also determine parasitism success (Dubuffet et al., 2007).

#### 4.2.3 Environmental effects

Different environmental conditions influence the performance of parasitoids. Two important stress factors are temperature (Delava, Fleury, & Gibert, 2016; Ris, Allemand, Fouillet, & Fleury, 2004) and insecticides (Cossentine & Ayyanath, 2017; Komeza, Fouillet, Bouletreau, & Delpuech, 2001; Rafalimanana, Kaiser, & Delpuech, 2002). Parasitism of *P. vindemmiae,* for example, was significantly negatively affected by Spinosad, a commonly used insecticide against *D. suzukii* (Cossentine & Ayyanath, 2017). Hence, releasing *P. vindemmiae* as biological control agent in insecticide‐treated fields might reduce its efficiency. Two biotic factors that can alter parasitization success are heritable viruses that manipulate the parasitoids’ biology (Martinez, Lepetit, Ravallec, Fleury, & Varaldi, 2016; Martinez et al., 2012) and competitor species exploiting the same host resource. The latter is especially relevant when applying a new biological control agent in an area where another parasitoid is already present as it may reduce the original agents’ efficiency. In contrast, additive (Herrick, Reitz, Carpenter, & O'Brien, 2008; Shapiro‐Ilan, Jackson, Reilly, & Hotchkiss, 2004; Snyder & Ives, 2003) or even synergistic interactions (Mesquita & Lacey, 2001) of the agent with other species are possible and might enhance the efficacy of the control agent.

#### 4.2.4 Implications for selection or selective breeding of a biocontrol agent

Of the indigenous parasitoids, the pupal parasitoids of *D. suzukii* appear to have the highest biocontrol potential, as they seem not to be affected by the high resistance level of the pest. Yet, *P. vindemmiae* and *T. drosophilae* have a relatively wide host range. This may cause high incidence of nontarget effects if released as control agent or low biocontrol efficiency against *D. suzukii* if they have higher preference for other host species. For example, the pupal parasitoid *P. vindemmiae* can parasitize more than 60 fly species, and is even able to hyperparasitize other (beneficial) parasitoids like *A. tabida* and *L. heterotoma* (Carton, Bouletreau, van Alphen, & van Lenteren, 1986; Marchiori & Barbaresco, 2007; Wang & Messing, 2004a; Zhao, Zeng, Xu, Lu, & Liang, 2013). The pupal parasitoid *T. drosophilae* has a smaller host range, but is still able to develop on numerous *Drosophila* species (Carton et al., 1986; Mazzetto et al., 2016). The use of those species as control agents, especially *P. vindemmiae*, therefore requires extensive assessment of ecological risks, intraguild predation, and potential effects on nontarget species (nontarget effects). Careful evaluation is needed to determine whether these risks outweigh the benefits. In case, it is deemed plausible to improve these species to become suitable biocontrol agents, an obvious trait that these species could be optimized for is to become more host‐specific. In fact, *T. Drosophilae* is already on the market as biocontrol agent in Italy, although its host preference and efficiency in colder conditions (e.g., <20°) (Rossi‐Stacconi et al., 2017) might need to be improved to increase its success rate and to be effective in northern countries (early in the season).

The only indigenous larval parasitoid with some parasitization success on *D. suzukii* is *L. heterotoma*. The virulence mechanism of *L. heterotoma* enables it to develop on a range of species of *Drosophila*,* Chymomyza,* and *Scaptomyza* (Eijs, Ellers, & Van Duinen, 1998; Fleury et al., 2009; Janssen, 1989), including *D. suzukii*. Whether their generalistic behavior is due to their venom load, venom composition or other factors is however not clear. Identifying the mechanism that enables at least some *L. heterotoma* to overcome host resistance of *D. suzukii* could be beneficial for the screening of individuals for specific traits for selection. Assaying proteins or specific alleles may be an efficient approach to select specifically for a trait of relevance for killing ability of the parasitoid.

In conclusion, the success of a control agent can be largely influenced by both genetic and environmental factors. For selective breeding, it is important to be aware of factors determining the agents’ performance in the field and during (mass) rearing as they are often different from experimental laboratory conditions. Important factors to investigate include the host‐finding ability of the agent in the field, phenotypic expression across abiotic conditions (reaction norm) and the nature of their interactions with other species in the field. Knowing these effects is important to predict their efficiency in the field and underlines the importance of assessing field experiments in the target area of release. In an interesting manner, additive or synergistic interactions of the control agent with other species can be exploited for biological control. However, the nature of their interaction (antagonistic or additive/synergistic) depends on, for example, timing (simultaneously or sequential) and rate of application (Hussein, Habustova, Puza, & Zemek, 2016; Shapiro‐Ilan et al., 2004).

## 5 STEP 4: IMPROVE AND DETERMINE THE SUCCESS OF THE PARASITOID

The large variation in parasitization success within natural enemies of *D. suzukii* can be exploited in different ways. The most straightforward method is by comparing strains and to choose one expressing optimal biocontrol trait values (Lommen et al., 2017). This however will not always yield the desired trait combinations, and further optimization is then required. This can be achieved by selective breeding or experimental evolution of an, preferably native, outbred strain or mixture of strains (e.g., to increase genetic variation). Populations present in the invaded area might also be crossed with those coevolved with the pest; however, their import and release might be slowed down by national and international regulations, including the before‐mentioned Nagoya Protocol (Hajek et al., 2016). Selective breeding and experimental evolution can increase the frequency of specific alleles, to express desirable trait values in the population of investigation. This has already been done successfully for centuries in livestock and plant breeding. Selective breeding and experimental evolution require substantial genetic variation of the trait(s) of interest and a large effective population size. Methods of selection are described in, for example, Kawecki et al. (2012), Garland and Rose (2009) and Lommen et al. (2017). They include exposing a population to experimental conditions to obtain a strain adapted to the specific environment (experimental evolution), and selecting and breeding only those individuals harboring the desired trait(s) (artificial selection). Agents can be selected either on phenotype, breeding value (sum of effects of all alleles of an individual) or on a single allele. The choice depends on the ability to measure phenotypic value, genomic knowledge and money available, and on the genetic architecture of the trait(s) of interest (i.e., whether one locus of large effect or many loci of small effect are selected). When the candidate agents’ genome is sequenced, genetic markers may assist artificial selection when variable genomic region(s) are identified that are associated with the target trait(s). Using these markers to select individuals for trait(s) of interest (marker‐assisted selection/genomic selection) can save time and increase accuracy of selection (Xu et al., 2012). Instead, or in addition, hybridization of different strains can increase genetic variation of the agent to be improved and/or may be a way to generate new genetic combinations, to alter the performance of the control agent. A few studies have demonstrated the potential of selectively breeding biocontrol agents (e.g., Hoy, 1985, 1986; Lommen, 2013), in particular parasitoids (e.g., Kraaijeveld, Hutcheson, et al., 2001; Rouchet & Vorburger, 2014; Weseloh, 1986). The relative short generation time and size makes (selective) breeding of insects more feasible compared to livestock and crops. In addition, knowledge about the genetic basis of target trait(s) could make optimization more efficient using molecular tools (e.g., markers) to rapidly select for certain trait(s) and predict the response to selection.

Two important drawbacks that can hinder the success of (selective) breeding are low genetic variation and adaptation to laboratory conditions (Hopper et al., 1993; Mackauer, 1976; Sørensen, Addison, & Terblanche, 2012). The amount of genetic variation depends on the starting population and (selective) breeding method. Selective breeding causes a decrease in variation as only a subset of the population with the desired characteristics is selected to contribute to the next generation. This results in higher chances of inbreeding depression and loss of (desirable) alleles and fixation of (deleterious) alleles due to genetic drift. In particular when is aimed for ongoing, long‐term control of an agent (one or several years), a decrease in genetic variation might limit their ability to respond to environmental changes within and between years. To limit inbreeding effects, the source(s) and size of the starting population should be chosen carefully to keep a large effective population size and thus large genetic variation (Lommen et al., 2017; Mackauer, 1976; Meuwissen & Woolliams, 1994). To retain genetic variation, breeding schedules are available that maintain large effective population size (e.g., Lommen et al., 2017; van de Zande et al., 2014). In addition, the selection regime and the intensity of selection influence genetic variation and should therefore be chosen carefully to maintain a fit population. In an interesting manner, in haplodiploid systems (including all hymenopterans parasitoids), in which males develop from unfertilized eggs and females from fertilized eggs, inbreeding depression is less prevalent. Deleterious recessive alleles that are expressed in males are rapidly purged by selection, thus reducing deleterious allele frequencies (Henter & Fenster, 2003).

Breeding and experimental conditions should preferably simulate natural conditions of the target area to enhance the agent's success rate and to prevent adaptation to laboratory conditions. Mass rearing can result in unintentional behavioral changes due to genotypic changes (selection) or environmentally induced changes (phenotypic plasticity, such as learning) (Chambers, 1977; Mackauer, 1976). Parasitoids reared on artificial diet can, for example, develop preference for an atypical artificial diet over its natural one (Bautista & Harris, 1997). In addition, detrimental behavioral alteration of biocontrol agents has been shown in dispersal ability, host searching and mating behavior (Boller, 1972; Chambers, 1977). A parasitoids’ host‐searching behavior is also influenced by learning of host (e.g., pheromones) and host‐habitat cues (e.g., shapes and substrate odor). Incorporation of stimuli during mass rearing may prevent behavioral changes to unnatural situations and increase its effectiveness in the field to localize and parasitize the pest (Boller, 1972; Giunti et al., 2015). Thus, also nonheritable variation can be exploited in the optimization process of strains, taking advantage of insights into the different factors that contribute to the phenotypic variation. This can be achieved by (a) mass rearing the agent on the pest and/or (b) exposing them to pest‐related cues of the habitat to be released in (Giunti et al., 2015). Therefore, although maybe practically and economically challenging, control agents of *D. suzukii* should preferably be reared on the pest itself and possibly on economically important fruits to increase and maintain their adaptation to the pest and pest habitat. Challenges include dietary restrictions, relative low fecundity, and relative high sensitivity to climatic conditions (Emiljanowicz, Ryan, Langille, & Newman, 2014; Hamby et al., 2016; Iacovone et al., 2015). This results in slower establishment and build‐up of laboratory population and more time and care to rear them (Iacovone et al., 2015; personal observations). However, with increasing knowledge on the fly's biology (e.g., Hamby et al., 2016) and culturing methods (e.g., Sampson et al., 2016; Young, Buckiewicz, & Long,^2018^), (mass) rearing is becoming more feasible. Once a large population has been established, it can be kept under the right laboratory conditions. In particular, the innate ability to find hosts, the ability to learn to localize hosts, and memory retention differ between parasitoid species and populations (van den Berg et al., 2011; Geervliet, Vreugdenhil, Dicke, & Vet, 1998; Koppik, Hoffmeister, Brunkhorst, Kiess, & Thiel, 2015; Perez‐Maluf et al., 2008; Smid et al., 2007). This should be taken into account by choosing candidate agents with high searching ability or targeting these traits for artificial selection, as, for example, done by van den Berg et al. (2011).

Quality control of a selected control agent is required to verify its improved performance for mass rearing and/or in the field (Lommen et al., 2017). In particular, the effect of phenotypic plasticity and correlated responses on the performance of the agent should be investigated. To determine the success of a control agent, (semi‐)field experiments should be performed with preferably the same pest population and under environmental conditions similar as in the target area(s) for release (e.g., crop type, climatic conditions, presence of alternative prey/hosts). Important factors to investigate are the control agents’ ability to kill the pest and reduce crop damage, the duration of the agent's effect (one or multiple generations) and the release method of the agent. In other words, its efficiency should be characterized in a variety of relevant conditions in time and space, preferably on large scale (phenomics, see Box 1), to determine in which conditions the agent can be used. The second factor influencing its success is correlated responses, meaning that selection on one trait might change the expression of other traits (Kraaijeveld, Limentani, & Godfray, 2001; Ueno, De Jong, & Brakefield, 2004). Trade‐offs, a beneficial change in one trait that is linked to a detrimental change in another, may be caused by genetic correlations (pleiotropic effects, genetic linkage) or resource allocation (Brakefield, 2003), and may decrease the fitness and efficiency of the control agent. Parasitoids selected for high counterdefenses, for example, showed a slower egg‐hatching rate, which might give them a fitness disadvantage during super‐ or multiparasitism (Kraaijeveld, Hutcheson, et al., 2001). Potential trade‐offs and its effect on the agents’ efficiency should therefore be investigated upon selection to secure the efficiency of the control agent.

## 6 CONCLUSION AND FUTURE RESEARCH DIRECTIONS

Finding and optimizing potential agents requires insight into natural variation of traits important for biological control and factors that determine this variation. To what extent, native natural enemies can be optimized by selective breeding depends on the genetic architecture of the target trait, the amount of genetic variation, and environmental constraints. These factors vary and should be determined for each individual case. Therefore, to systematically develop successful control agents, we propose a four‐step approach to exploit intraspecific variation efficiently (Figure 2). We have illustrated this optimization strategy with an example of killing efficiency of parasitoids of the new invasive pest *D. suzukii*. We conclude that there is large variation in killing efficiency and field performance within and between parasitoid species. As this trait seems, at least in part, to be determined by genetic factors and previous research has shown feasibility to increase the killing ability of parasitoids through selective breeding (Kraaijeveld, Hutcheson, et al., 2001), indigenous parasitoids of *D. suzukii* might be optimized for biological control. In particular, the pupal parasitoid *T. drosophilae* and larval parasitoid *L. heterotoma* might be subject to improvement in Europe and North America. Before setting up efficient breeding programs for these candidate species, additional field explorations are needed for exploring amounts of intraspecific variation to choose and/or use the most competent strain(s). Besides killing efficiency, other traits can be targeted for optimization, such as host range (in particular for pupal parasitoids) to increase environmental safety, and fecundity to increase mass rearing efficiency. In an interesting manner, traits important for biocontrol (Table 1) could also be of interest for breeding insects for use in sterile insect technique (SIT) and for feed and food production.

More potential agents might be found with increasing residence time of the pest in the invaded area. The number of indigenous species able to kill *D. suzukii* is almost 70% lower than in the pest's native range. However, there are at least some parasitoid species that seem to be able to cope to some extent with the invasive pest, such as *L. heterotoma* and *T. drosophilae* in Europe and North America. The potential of these parasitoids to naturally adapt to the high resistance of *D. suzukii* is more likely when they encounter this host frequently. Adaptation to *D. suzukii* might give certain species a fitness advantage as it is an underexploited ecological niche within the local ecosystem. However, it is difficult to predict the time frame in which this would occur.

Optimizing control agents requires thorough understanding of which traits significantly enhance their performance. The assessment of biocontrol traits and predicting optimal expression values, however, are complicated as laboratory results do not always hold in nature. In addition, no list of optimal trait values exists because these may differ with pest species, the crop to protect, climatic conditions of target area, release method (long‐term vs. short‐term control), and target area (greenhouse, small or large orchard) (Lommen et al., 2017; Wajnberg, 2004). Identification of important biocontrol traits for specific pests and target areas or finding a generic approach for their identification could be highly beneficial for the efficiency of biocontrol (“personalized biocontrol”). Large‐scale phenotypic data collection (phenomics, see Box 1) could be an effective method to accomplish this. In addition, biological control could greatly benefit from genomic research as it can speed up and increase the success of selective breeding of natural enemies. Whole genome sequencing can aid the identification of genetic markers for marker‐assisted selection (Dekkers & Hospital, 2002), or to generate high‐resolution SNP maps to investigate the genetic architecture of relevant traits. To date, genetic data on biocontrol agents are often limited as genotyping costs are often too high for companies that are mass‐producing biocontrol agents (Lommen et al., 2017). With decreasing costs, it may become more feasible in the future.

## CONFLICT OF INTEREST

None declared.

## DATA ARCHIVING

No data are associated with this manuscript.
